# Internationalization of Traditional/Complementary Medicine products: market entry as medicine

**DOI:** 10.1186/s13020-018-0209-6

**Published:** 2018-10-11

**Authors:** Jiatong Li, Jianfan Zhu, Hao Hu, Joanna E. Harnett, Chi Ieong Lei, Ka Yin Chau, Ging Chan, Carolina Oi Lam Ung

**Affiliations:** 1 State Key Laboratory of Quality Research in Chinese Medicine, Institute of Chinese Medical Sciences, University of Macau, Taipa, Macao; 2 The University of Sydney School of Pharmacy, Faculty of Medicine and Health, The University of Sydney, Sydney, Australia; 3 Pharmaceutical Society of Macau, Taipa, Macau; 4 City University of Macau, Taipa, Macau

**Keywords:** Traditional Chinese medicines, Chinese patent medicines, Proprietary Chinese medicines, Traditional and Complementary Medicine, Registration, Internationalization, Quality, Safety, Efficacy

## Abstract

Internationalization of Traditional/Complementary Medicine (T&CM) products is important for initiating and sustaining developments in this field. Particularly for traditional Chinese medicines (TCMs), the global market continues to expand due to an interest in the potential clinical benefits of traditional approaches that are largely considered lower risk and lower cost than many conventional treatments. While the benefits of internationalization hold clear advantages for the business of T&CM products, keeping abreast of regulatory processes in different countries and regions that regularly revise market entry requirements is challenging. At present, the regulations of T&CM products are country specific and largely based on a risk-based assessment with a focus on protecting the consumer. To date, systematic analysis of these regulatory differences between countries and regions is limited. Publically available information about the legal requirements for the market entry of T&CM products were obtained from the relevant regulatory authority’s websites for selected countries and regions (Macau-China, Hong Kong-China, Singapore, Australia, Canada, the European countries and the US). The market entry requirements in terms of quality, safety and efficacy of T&CM products for each country were analyzed and compared. Major differences were identified in the classification of T&CM products, market entry pathways, requirements of compliance with Good Manufacturing Practices; and level of evidence to demonstrate safety and efficacy based on historical use, non-clinical and clinical studies. Variations in the evaluation standards adopted by regulatory authorities pose a number of barriers and opportunities for the internationalization of T&CM products and have great implications for internationalization of TCMs from the sponsors’ and the regulators’ perspectives.

## Background

Chinese Medicine is considered a traditional medicine practice and a popular approach to health care adopted by many individuals throughout the world [1–4]. It refers to both traditional Chinese medical practices and traditional Chinese medicines (TCMs) [4]. In China, an ageing population, increased prevalence of chronic conditions, a rising GDP, and accommodating medical insurance programs are all considered drivers of the a growing TCMs market [5]. This has prompted the Chinese government to identify Chinese Medicine as a priority and they have issued supportive measures to support Chinese Medicine in a strategic attempt to provide universal health coverage. The State Council in the 13th Five-Year Plan (2016–2020) aim to ensure all citizens are covered with health care by 2020 and that Chinese Medicine will be an important element in achieving that goal and further supported with scientific research, education and cultural influences [6]. In 1996, China introduced the concept of “internationalization of TCMs” which had two main objectives: (1) to achieve the sustainable development of TCMs through fostering international trade and expanding TCMs in the global pharmaceutical market; and (2) to facilitate the development of appropriate regulatory systems that allows reasonable market entry under the protection of the local laws and regulations [7].

In the past 2 decades, advancements in research technologies has enabled a substantial body of research in TCMs to be conducted [8]. Research of TCMs supported with advancement in technology has better informed our understanding of TCMs, through the identification of active constituents and mechanisms of actions, and the identifying the forms and doses required to achieve positive therapeutic outcomes and minimize side effects and toxicity [9]. Despite this progress in developing the evidence base, the internationalization of TCMs has faced both intrinsic and extrinsic challenges. Take Chinese patent medicines (CPMs, referring to any TCMs formulated into a finished dosage form) as an example. Like any other types of TCMs, CPMs have uniquely complicated features [10]. They are used based on Chinese Medicine theory which cannot be fully explained or comprehended within the language or context of modern science. Maintaining a consistent constituent profile for many CPMs is difficult due to the lability of some constituents during the production process [8]. The quality control of CPMs is also difficult to sustain with challenges in accurate plant identification, cultivation, harvesting and the processing of herbal materials. Extrinsic factors including pesticide residues, and contamination with heavy metal and other unwanted chemical are regularly encountered during the production of CPMs. To deal with some of these challenges, the development of specifications for quality control of end products is under development. In contrast to pharmaceutical drugs, CPMs are inherently more complex to evaluate due to the complexity of multiple constituents and diverse therapeutic actions, and being less commonly the subject of large randomized controlled trials (RCTs).

One of the biggest extrinsic challenges facing the internationalization of TCMs is the registration/policy barriers [3, 5, 10–12]. Firstly, the terms used to refer TCMs varies from country to country. Depending on the culture, TCMs may be known as traditional medicines or complementary medicines (referred to the broad set of health care practices that are not part of the country’s own tradition or conventional medicine but used in additional to conventional medicine) [13]. More than 90 countries and regions have national polices, laws and regulations for the marketing approval of Traditional and Complementary Medicine (T&CM) products including TCMs [3]. However, depending on the national situation, the criteria for marketing T&CM products varies across the countries indicating that regulatory authorities have different standards and/or interpret evidence for quality, safety (risk) and efficacy, therefore the possible risk of using such products differently.

Some countries, as reported by the World Health Organization (WHO), have national policies on T&CM products and measures to ensure the quality, safety and efficacy of the T&CM products with a respect for the uniqueness of the traditional modality [4]. For some other countries, the regulatory authorities may include T&CM products into the scope of drug regulation, requiring that T&CM products meet the same set of stringent registration requirement as for other pharmaceutical products for marketing approval [14]. In some cases, authorities have additional regulations and guidelines to supplement the drug legislations to address issues related to the safe and appropriate use of T&CM products. There are also cases where T&CM products, when deprived of certain health claims, may be marketed as “non-medicine” entities. As a result, eligible T&CM products may be marketed as “medicine” only in some countries and areas depending on the regulations [3, 14].

With due respect to the therapeutic values of T&CM products (particularly TCMs), the identity of “medicine” in any regulatory systems is sought after during the course of internationalization. Currently, little research has been conducted to systemically analyze the regulation standards of T&CM products as a “medicine” entity across the countries. Therefore, the objective of this study was to compare the regulatory process for T&CM products using CPMs as products of interests among selected countries and regions. Specifically, the requirements for assessing quality, safety and efficacy for T&CM products classified as ‘medicine’ were analyzed. The findings of this study will be useful to inform a global perspective on the regulations of T&CM products, and specifically the internationalization strategies for TCMs.

Countries and regions including Macau-China, Hong Kong-China, Singapore, Australia, Canada, the European countries and the United States (US) were selected for this study based on the following considerations: (1) they have established regulatory practices that assess T&CM products as medicine; and (2) they represents a range of regulatory systems for T&CM products. A systematic qualitative analysis was conducted and included the review and interpretation of official legal documents related to the marketing of T&CM products. The publicly available documents were identified from the official website of the respective governing authorities of the selected countries and regions: Health Bureau, The Government of the Macau SAR (http://www.ssm.gov.mo) [15], Chinese Medicine Council, The Government of the Hong Kong SAR (http://www.cmchk.org.hk/) [16]; Chinese Medicine Division, Department of Health, The Government of the Hong Kong SAR (http://www.cmd.gov.hk/html/eng/index.html) [17], Health Sciences Authority of Singapore (http://www.hsa.gov.sg) [18], The Therapeutic Goods Administration of Australia (http://www.tga.gov.au) [19], Health Canada (https://www.canada.ca/en/health-canada/services/drugs-health-products/natural-non-prescription.html) [20], European Medicines Agency (http://www.ema.europa.eu/ema/) [21], Medicines and Healthcare Products Regulation Agency of the UK (https://www.gov.uk/government/organisations/medicines-and-healthcare-products-regulatory-agency) [22], Medicines Evaluation Board of the Netherlands (https://english.cbg-meb.nl/) [23], and US Food and Drug Administration (https://www.fda.gov/AboutFDA/default.htm) [24]. The terminology used to describe the regulatory framework for marketing T&CM products and evidence requirements for demonstrating quality, safety and efficacy of the product in each regulatory system was extracted, interpreted and analyzed.

## Macau-China

Macau is one of the two special administrative regions (SAR) of the People’s Republic of China, located on the western bank of the Pearl River Delta in the southern Guangdong Province. Macau had been a Portuguese colony since the mid-16th century until the handover to China in 1999 when it became a SAR. The city exercises a high degree of autonomy under the principle of “One country, two systems” and has a local regulatory system for TCMs which covers CPMs. Due to Macau’s deep-rooted connections with Portuguese-speaking countries, Macau is strategically placed for the facilitation of TCMs internationalization.

The regulation of CPMs and other TCMs is overseen by the Department of Pharmaceutical Affairs, Health Bureau [15]. At the moment, Macau does not have a registration system for CPMs. However, an alternative process is in place that takes reference of the regulatory decision made by the authority of the country of origin. Therefore a “sales pre-approval system” is adopted and supported and supplemented by a number of legislations, regulations and technical instructions. *Decreta*-*Lei n 53/94/M*, enacted in 1994, is the main legislation governing the licensing and operating conditions of companies involved in the import, export and wholesale of TCMs, as well as the TCMs pharmacies. Although it does not cover the sales pre-approval requirements of TCMs, it gives the legal definition of TCMs which refers to any medicines, plant or animal ingredients, or any materials extracted from these ingredients, used according to Chinese Medicine and Chinese pharmacology for the prevention or treatment of diseases, or the regulation of bodily functions.

In order to better inform the safety and efficacy of TCMs, a “List of Traditional Chinese Medicine Ingredients for consumption in Macao SAR” was developed by the Health Bureau, which consists of three sub-lists: (1) Part I—toxic traditional Chinese materials (30 types); (2) Part II—common therapeutic traditional Chinese materials (562 types); and (3) Part III—Chinese medicinal materials that are also used as food (112 types). According to *Technical Instruction No 02/2005*, CPMs refers to a product which has a definite pharmaceutical form and composes of (1) one or more ingredients from Part I and/or Part II; (2) one or more ingredients from Part III and carry claims about treating, alleviating or preventing a disease or symptoms on the product label; or (3) formulated with one or more natural medicinal ingredients from plants, animals, or minerals under the guidance of traditional Chinese Medicine theory, and are applied to the human body for the purpose of treating, alleviating, or preventing diseases or symptoms thereof. Sales of CPMs are exclusive to TCMs pharmacies. For products composing ingredients from Part III but without any claims of disease/symptom treatment, alleviation or prevention, they will not be considered as CPMs and not subject to sales restrictions.

The local manufacturing capacity of CPMs is very limited. Most of the CPMs available in the market are imported products. According to the “sales pre-approval system”, prior to importation of CPMs by licensed importers, certain documents must be submitted which includes a Drug Registration Certificate issued by the governing authority in the country of origin, certificates of analysis (including microbial and heavy metal contamination) for each batch of CPMs and the manufacturer’s license to demonstrate the minimal quality, safety and efficacy assurance. The adherence to GMP is recommended but not mandated. The maximum limits of heavy metal and toxic elements allowed in CPMs are specified in *Despacho No. 10/SS/2013*. *Technical Instruction 01/2004* outlines the microbiological limits for different dosage forms of TCMs. When the CPMs originate from a country with a registration system in place, test reports of microbiological limits, heavy metals and toxic element content may be exempted. Labelling requirements stipulated in *Technical Instruction 04/2005* apply to all TCMs.

Additional regulations and guidelines are in place for specific TCMs as a safeguard. For TCMs which contain high-risk Chinese medicinal materials, either a banning system or a restricted-use system is in place. For instance, *Radix Aconiti Lateralis Preparata*, which is an ingredient from Part II of the “List of Traditional Chinese Medicine Ingredients applied in the Macao SAR”, is recommended to be processed and used according to specific instructions with precautions due to the potential risks of toxicity as indicated in the official document “Instructions of using *Radix Aconiti Lateralis Preparata*”. Similar instructions are also in place for *Rhaponticum uniflorum* (L.) DC. Due to the high risk of toxicity, according to the *Health Bureau Director Decision No. 6/SS/2004*, utilization, manufacture, importation and sales of *Caulis Aristolochiae Manshuriensis* (Guanmutong), *Radix Aristolochiae Fangchi* (Guangfangji) and *Radix Aristolochiae* (Qingmuxiang) are prohibited in Macau. Additional document requirements are applicable to a special CPMs, Niuhuang Jiedu Tablets, as stipulated in the *Chief Executive Decision No. 132/2001*. In order to minimize the risks associated with bovine-derived drugs, additional production and importation requirements of bovine-derived drugs are listed in the *Chief Executive Decision No. 120/2005* and *Technical Instruction No. 02/2001*.

As stated by the Macau SAR Government, the draft regulations for the registration scheme for TCMs have been prepared by the Health Bureau, and the relevant legislative processes are underway.

## Hong Kong-China

In Hong Kong, traditional Chinese Medicine is widely accepted as playing a substantial role in the public healthcare sector. To tighten the regulation of Chinese Medicine practice and Chinese herbal medicines, with a view to safeguarding the public, the Chinese Medicine Ordinance (Cap.549) was enacted in 1999, and officiated the legal status of TCMs. Accordingly, proprietary Chinese medicines (pCms) must be registered by the Chinese Medicines Board of the Chinese Medicine Council of Hong Kong (the Council) before they can be legally marketed [16]. The Council is a statutory body established under the Chinese Medicine Ordinance responsible for formulating and implementing regulatory measures of TCMs. The Council receives professional and administrative support from the Chinese Medicine Division of the Department of Health which is responsible for the enforcement of Chinese Medicine Ordinance [17].

By definition in the Chinese Medicine Ordinance [25], pCms refer to any proprietary product: (a) composed solely of the following as active ingredients: (i) any Chinese herbal medicines, (ii) any materials of herbal, animal or mineral origin customarily used by the Chinese; or (iii) any medicines and materials referred to in subparagraphs (i) and (ii) respectively; (b) formulated in a finished dosage form; and (c) known or claimed to be used for the diagnosis, treatment, prevention or alleviation of any disease or any symptom of a disease in human beings, or for the regulation of the functional states of the human body. For registration purposes [26], CPMs from China may fall into one of the three categories for pCms in Hong Kong: Established Medicines (pCms that are formulated according to an ancient prescription, a modified ancient prescription, a pharmacopoeia prescription or other prescriptions originated from the National Drug Standards of the People’s Republic of China), Non-established Medicines (pCms that are used for the purpose of regulating the functional states of the human body or Single Chinese medicine granules) and New Medicines (pCms that contain newly discovered Chinese herb or new ingredient, or have new indication, altered route of administration or altered dose form).

Any of these three categories may be subject to registration approval: Group I, Group II and Group III. Different registration groups mainly differ in the registration requirements and documents related to the safety and efficacy of the pCms. Group I and Group II registration groups have different documentary requirements for the mutagenicity, carcinogenicity and reproductive toxicity of the pCms. Group III registration requirements are the most stringent as they requirement full documentation to demonstrate safety and efficacy of the New Medicines.

### Quality

The documentary requirements to demonstrate the quality of pCms are the same for Group I, Group II and Group III registration dossier. For the quality evaluation of pCms, manufacturing method, physicochemical properties of crude drugs, product specification, method, and certificate of analysis are assessed. As for stability of the pCms, Group I registration dossier composes of either an accelerated stability test report or general stability test report, but for Group II and Group III registration dossier, a real-time stability test report is essential. The manufacturers must be licensed to manufacture pCms. They are required to comply with all aspects of GMP as stipulated by the Hong Kong Good Manufacturing Practice Guidelines for Proprietary Chinese Medicines.

### Safety

In order to demonstrate the safety of pCms, all of the three registration groups require the submission of a certificate of analysis that reports the test results for heavy metals and toxic chemicals, pesticide residues and microbes, acute toxicity data, long-term toxicity data and a summary report outlining the product safety. For pCms to be administered on the skin or mucous membrane, additional requirements including reporting on the topical application toxicity is required for all three registration groups. For Group II and Group III registration groups, a report on the mutagenicity, carcinogenicity and reproductive toxicity are required for pCms which contain established or new ingredients that are or suspected to be cytotoxic, carcinogenic or mutagenic, or relates to pregnancy or, proven to have toxic effects on reproductive system.

### Efficacy

In order to demonstrate the efficacy the pCms, information about interpretation and principle of formulating a prescription (except for pCms are single Chinese medicine granules), as well as reference materials on the efficacy are needed. The major discrepancies in the requirements in the thee registration groups lie on the principal pharmacodynamic study reports, general pharmacological study reports, and the clinical trial protocol and summary report which are essential only to Group III registration. In other words, pCms which are not classified as New Medicines may have the health claims or indications supported with referencing materials only.

## Singapore

The Health Science Authority (HSA) is responsible for the drug regulatory work, including the issuance of the licenses of the importers, wholesalers, manufactures and re-packers of Chinese proprietary medicines (Cpm) [18]. It is a statutory board of Singapore Ministry of Health that aims to protect and advance national health and safety. The Chinese Proprietary Medicines Unit is one of the divisions of HSA specifically responsible for the administration of regulatory control and approval of Cpm. Cpm that is intended to be imported and sold must be registered for product listing approval. In accordance with Medicines (Traditional Medicines, Homoeopathic Medicines and other Substances) (Exemption) Order in Medicine Act 1985 (CAP 176), Cpm refers to any medicinal product (a) which has been manufactured into a specific dosage form and contains one or more active substances derived wholly from any plant, animal and/or mineral using active substances that are listed in the current edition of “A Dictionary of Chinese Pharmacy” or “The Chinese Herbal Medicine Materia Medica”. Injectable products or medicinal products containing chemically-defined isolated constituent of any plant, animal or mineral, or any combination thereof are not considered Cpm. The number of the approved Cpm increased considerably from 2076 in 2011 to over 10,000 in 2013.

### Quality

Cpm in Singapore can be manufactured either locally or overseas. In order to ensure the Cpm are consistently produced with the quality standards, GMP guideline must be complied by all Cpm manufacturers and assemblers in Singapore in line with the Health Products Act 2007 and the Medicines Act 1985. Singapore is a member of PIC/S, thereby the PIC/S Guide (Guide to Good Manufacturing Practice for Medicinal Products Part I and PIC/S GMP Guide (related annexes) are implemented for manufacturing or assembling Cpm to assure the quality of those products. When assessing registration application for Cpm, audits will be conducted to the Cpm manufacturers and assemblers to ensure adequate quality and safety. Overseas Cpm manufacturers and assemblers also need to provide the GMP certificate (if any). According to the Medicines (Prohibition of Sale and Supply) Order, Cpm in question also needs to undergo tests for toxic heavy metals, microbial contamination, TSE (only for products containing materials derived from ruminants) and fermented substances (only for products containing fermented substance such as Cordyceps, Red Yeast Rice). Extra requirements apply if the Cpm contain substances listed in the Endangered Species (Import & Export) Act 2008. The test parameters and methods of the finished Cpm should be developed in consultation of the latest edition of the British Pharmacopoeia, Chinese Pharmacopoeia, European Pharmacopoeia, United States Pharmacopoeia, etc. especially for the toxic metals and microbial contamination.

### Safety

A declaration stating the absence of any poisons as defined in the Poisons Act 1999 (Cap. 234) (i.e. Amygdalin, pangamic acid or its salts, danthron, suprofen or its salts and rhodamine B) is required for the import of Cpm. According to the same Act, the naturally occurring poisons in Cpm, i.e. ephedra alkaloids, lovastatin, sodium borate, lobelia alkaloids and aconite alkaloids, and those substances considered as high-risk (e.g. those with slimming claims) must be tested and verified to be within the acceptable limits at local or overseas laboratories with accredited testing methods. Test reports must be compiled within 2 years from the evaluation date of the Cpm.

### Efficacy

These market entry requirements focus on the safety and quality of the Cpm rather than efficacy as no clinical efficacy data is required for the registration application. The indications can be claimed in terms of TCMs system of therapeutics, historical records and traditional uses outlined in pharmacopeias or with reference to A Dictionary of Chinese Pharmacy and The Chinese Herbal Medicine Materia Medica. However, there are restrictions on the claims as the labels, packaging materials and package inserts of Cpm cannot indicate any of the 19 diseases/conditions in accordance with those specified in the First Schedule of the Medicines Act. These conditions include blindness, cancer, cataract, drug addiction, deafness, diabetes, epilepsy or fits, hypertension, insanity, kidney diseases, leprosy, menstrual disorders, paralysis, tuberculosis, sexual function, infertility, impotency, frigidity and conception and pregnancy.

## Australia

In Australia, Chinese medicine herbal products (equivalent to CPMs in this study), together with other herbal medicines, vitamin and mineral supplements, nutritional supplements, and other traditional remedies, fall into the category of complementary medicines (Therapeutic Goods Act 1989) [19, 27]. ‘Complementary medicines’ are regulated by the Therapeutic Goods Administration (TGA) and are defined as ‘therapeutic goods consisting wholly or principally of one or more designated active ingredients, each of which has a clearly established identity and (a) a traditional use or (b) any other use prescribed in the regulations’. The TGA has the responsibility for administering the federal Therapeutic Goods Act 1989, which provides the national framework for the regulation of therapeutic goods, including complementary medicines in Australia.

The regulatory framework for complementary medicines is based on a risk based system. The TGA currently takes a two-tiered approach based on risk: (1) Listed medicines or (2) Registered medicines. To be approved as a listed complementary medicine, the product is restricted to certain low risk ingredients in acceptable amounts that are permitted for use by the TGA and can only make indications for health maintenance and enhancement and/or for non-serious self-limiting conditions. Registered medicines can carry more specific therapeutic claims and/or contain a herb with a higher risk profile. In addition, registered medicines are independently assessed for quality, safety and efficacy. Registered CPMs on the Australian Register for Therapeutic Goods (ARTG) are currently outnumbered by listed CPMS. In 2016/2017, 1581 new complementary medicine products were listed on the ARTG as compared to 34 new registered complementary medicines. Listed medicines carry a unique AUST L number.

### Quality

The same level of quality assurance mechanisms in terms of GMP requirements apply to all the products in the two-tiered regulatory system of Australia. As such, all the medicines regulated by the authority must be made in TGA approved manufacturing facilities. The TGA requires compliance with the Pharmaceutical Inspection Co-operation Scheme (PIC/s) Code of Good Manufacturing Practice for medicinal substances and provides interpretive guidelines for the particular requirements of complementary medicines on supplier qualification, stability testing, product quality reviews, sampling and testing, and process validation. Australian and overseas manufacturers are assessed prior to supply of complementary medicines either through regular on-site inspections or, for manufacturers with evidence of GMP compliance available from recognized regulators, compliance verifications (paper-based assessments). Assessment conducted by the TGA includes, but is not limited to, information about the manufacturing process and controls. This requires evidence of the validation of processes, testing reference materials, test procedures and the results for the analysis of product strength, purity and stability. An application for a GMP clearance by an off-shore manufacturer may be made on the basis they are regulated by the manufacturing country’s governing authority and are recognized by the Mutual Recognition Agreement.

### Safety

Listed complementary medicines must only contain ingredients that have been approved by the TGA as being of low risk, with limits on quantities where relevant, and must not make high-level claims. For new and innovative ingredients, it is required to apply for the evaluation of the substance for its inclusion on the TGA’s list of permissible ingredients. This process may take approximately 1–2 years for the application to be evaluated which is a significant investment of time and money. The number of new listed medicine ingredients in 2016/2017 was 79, most of which are ingredients that were made available for excipient use in specific circumstances in listed medicines [28]. The TGA makes an assessment about the safety of an ingredient as it relates to therapeutic claim being made and associated safety warnings.

### Efficacy

The TGA regulates the claims about efficacy on the basis of the two tiered system as described above. For claims like “treats, cures or prevents a disease” or “treats vitamin/mineral deficiencies”, it is considered a high level efficacy claim and the product would require being assessed for a registered status i.e. AUSTR#. Whereas lower level claims of efficacy like “health enhancement”, “reduce the frequency of an event”, “assists in management of a disease”, “health maintenance”, or “vitamin or mineral supplementation” are considered medium or general level. In this case, it is the responsibility of the sponsor to ensure they hold evidence to support such claims. There are no requirements for the sponsors to provide efficacy data to the TGA prior to or during the pre-marketing stage but the sponsors must certify that efficacy data is available on request. Traditional evidence is also taken into consideration for low risk claims of efficacy and is based on respected Pharmacopoeias, Materia Medica and herbal monographs documenting human use of over three generations, equating to approximately 75 years.

### Recent development

It has been proposed that the current two-tiered regulatory framework is further developed to include a third or middle tier [29]. This will involve introducing a new product assessment pathway that will sit between the existing listed medicine (low risk) and registered medicine (high risk). Furthermore, a list of permitted indications which must be used by the lowest risk complementary medicines will be developed to further restrict the claims used and allowing sponsors to claim that their medicine has been independently assessed by the TGA for efficacy and that the product has undergone pre-market evaluation by the TGA. This regulatory reform aims to incentivize innovation within the complementary medicines sector. This presents an opportunity for data protection and market exclusivity if a product claims are based on new scientific evidence. In addition the proposed reform will improve transparency regarding the evidence for efficacy and allow consumers to make better informed decisions about the use of complementary medicines in their health care [27, 28].

## Canada

In Canada, T&CM products may be considered and regulated as Natural Health Products (NHPs) and are regulated by the Health Canada (HC) according to the Food and Drugs Act and the Natural Health Product Regulations [20]. Although there is no implication of “medicine” nature by the name, NHPs, by definition, refers to herbal or medicines of traditional medicine practices intended for use in the diagnosis, treatment, mitigation or prevention of a disease, disorder, or abnormal physical state or the symptoms thereof. According to the Natural and Non-prescription Health Products Directorate (NNHPD), NHPs are restricted to oral, topical, or sublingual routes of administration. The intended uses of NHPs encompass a wide range of claims ranging from maintaining or promoting health to diagnosis, treatment, prevention, and symptomatic relief of some diseases. Compared to the regulatory systems mentioned above, the health claims for NHPs in Canada are allowed to a greater extent and yet overlaps with mild claims of other non-medicine entities such as dietary supplements.

There are two subcategories to the NHPs claims: traditional health claims and modern health claims. TCMs, for instance, which contain multiple ingredients and are used based on a single cultural system of traditional medicine, Chinese Medicine, are considered NHPs with traditional health claims. For other T&CM products whereby the intended uses are not based on any one traditional medicine system, such claims are considered modern health claims. NHPs, when authorized for sale in Canada, are issued a product license and a Natural Product Number (NPN).

### Quality

The quality of NHPs focus primarily on characterization, identification, quantification and purity. For this, quality requirements including specific standards for chemicals, processed ingredients and extracts; compliance with the Good Agricultural and Collections Practices Guidelines; assay of the botanical ingredients, isolates or synthetic duplicates, live microorganisms and enzymes; microbial contaminants testing; chemical contaminant testing such as heavy metals, mycotoxins, solvent residents, pesticides, antibiotics, etc. are mandated in the “Quality of Natural Health Products Guide” published by the HC. Sites responsible for conducting manufacturing, importing, packaging or other activities in Canada must comply with the Good Manufacturing Practice standards in order to obtain a site license.

### Safety

The NNHPD has developed a collection monographs which can be used to support the safety and efficacy of a NHPs. These monographs are a comprehensive review of scientific data and information about a medicinal ingredient or multiple ingredients with certain health claims, containing all the information required for marketing approval. The “Traditional Chinse Medicine Ingredients” monograph, for instance, contains more than 300 TCM ingredients with indications of the specific conditions which the ingredients can be used for. NHPs conforming any of the monographs are likely to see expedition of the authorization process and consistency in the labeling of the products in the market. The evidence requirements for demonstrating safety depend greatly on the product’s intended uses indicated in the product claims. For medicinal ingredients, “benefit to risk” assessment forms part of the registration dossier which listed out the potential risks of the products in terms of severity and seriousness, probability or frequency, and other inherent risks of each of the ingredients. For non-medicinal ingredients, safety evidence may also be required. In either cases, whenever uncertainties about the risks are noted, additional evidence may be requested to allow thorough assessment by the authority,

### Efficacy

The therapeutic claims of NHPs must be supported with sufficient controlled clinical evidence of the ingredients generated from studies of which the protocols authorized by the Nonprescription and Natural Health Products Directorate. While monographs may be used to support “traditional health claims” or “modern health claims”, “traditional health claims” must be supported with evidence of at least 50 consecutive years of traditional use within a cultural health system or paradigm. Further to the safety document requirements, based on the three risk categories described in the “Management of Product Licence Applications (PLA) for Natural Health products” policy, different levels of evidence for premarket risk evaluation are required: Class I category refers to “low risk” products and attestation to a monograph is required to obtain marketing approval; Class II category refers to “medium risk” products and marketing application must be supported partly with the monograph and additional information such as phase 2 clinical trials to justify the novel features of the product; and Class III refers to “high risk” products whereby available information is limited and high level of evidence such as data from controlled clinical trials are required to obtain marketing approval. In cases of comparative therapeutic claims, head-to head clinical trials may also be required.

## European countries

In European countries such as the UK and the Netherlands [22, 23, 30, 31], the marketing pathways of T&CM products can be either traditional herbal registration (THR) or marketing authorization (MA) via simplified registration for T&CM products as stipulated in the European Directive 2004/24/EC serving as an amendment of Directive 2001/83/EC. Either THR or MA mandates the marketing of T&CM products to be granted based on legislation applicable for all EU countries in the European Economic Area. However, the directive also sees slight variations when implemented in each country as an adaption to the country’s own law [32–35].

The UK use the term herbal medicine to describe T&CM products including CPMs. According to the Human Medicines Regulation (2012), a herbal medicine refers to a product if the active ingredients are herbal substances (reduced or powdered, a tincture, an extract, an essential oil, an expressed juice or a processed exudate) and/or herbal preparations. On the other hand, T&CM products can be referred to herbal medicinal products in the Netherlands. By definition, herbal medicinal products, also referred to as phyto-therapeutic products, are medicinal products whose active ingredients contain exclusively plants, parts of plants or plant materials or combinations thereof, in a crude or processed form. Herbal medicines in the UK are regulated by the Medicines & Healthcare products Regulatory Agency (MHRA) whereas traditional herbal medicinal products in the Netherlands are regulated by the Medicines Evaluation Board.

Unlike the strict centralized marketing authorization applications of the medicines from European Medicines Agency, the decentralized simplified registration procedure which was officially promulgated in 2004 offers a simplified application pathway especially for the T&CM products with a longstanding historical use (used for at least 30 years, including at least 15 years within the EU), and hence provides an opportunity for T&CM products to enter the EU market in an expedited manner. The UK introduced the THR system for T&CM products relatively recently based on the EU Directives. In the Netherlands, presently only two traditional herbal medicinal products from China have been successfully licensed via these simplified procedures and marketed (*Diao Xin Xue Kang capsules* from Di Ao Group in 2012 and Sichuan, *and Danshen Capsule*s from Tasly Holding Group in 2016) [36, 37]. In UK, the Phynova Cold and Flu Relief Powder for Oral Solution was the first CPMs approved to be marketed in the UK market as a herbal medicine to treat colds.

### Quality

Quality of the medicinal products is considered the most important element in the simplified procedures of the application process. The authority requires the submission of evidence of GMP and QC tests for evidence of quality. The quality of the herbal substances, herbal preparations and finished product are required for inspection. For the quality of herbal substance, the Guideline on Good Agricultural and Collection Practice for Starting Materials of Herbal Origin (EMEA/HMPC/24618/2005) must be followed to assure the quality of the herbal substances from the cultivation in the wild, harvest and collection, to primary processing. In particular, detailed descriptions of the plants from which the herbal substances originate are required: botanical characteristics, phytochemical characteristics, toxic constituents, biological/geographical variation, cultivation/harvesting/drying conditions, and pre/post-harvest chemical treatments. The manufacturing site and process must comply with the GMP standards. For the quality of finished products, the manufacturing process, in-process controls and linking specifications, water content, impurities, toxic (heavy) metals microbial limits, mycotoxins (aflatoxins, ochratoxin A), pesticide, fumigation agents will be carefully assessed. The test methods and parameters must be developed with reference to the European Pharmacopoeia or specific monographs. Assays for the contents of the constituents of known therapeutic activity or active markers of the herbal preparations should be determined. Quality tests specific to the dosage form are also applicable.

### Safety

Unlike the full marketing authorization procedure, the simplified procedures do not require the submission of the full documentation on safety test and clinical trials to demonstrate safety and efficacy. Bibliographic data or toxicological tests to demonstrate safety may be acceptable. According to Article 16c (1) d of Directive 2001/83, safety should be justified by “a bibliographic review of safety data together with an expert report”. The prerequisite of longstanding use of the products has substantiated the safety of the traditional herbal medicinal products without the need of safety test. However, in case there are concerns about the safety of a traditional herbal medicinal product, the marketing authorization holder will have to provide comprehensive data upon the request from the authority.

To provide further guidance on safety assurance of the traditional herbal medicinal products, a number of scientific guidelines have been introduced such as EMEA/HMPC/138139/2005 (allergenicity issues of soya and peanut protein containing products). The establishment of Community herbal monographs or entries to the Community “List of herbal substances, preparations or combinations thereof for use in traditional herbal medicinal products” can be used to identify the new safety aspects of the listed plants, and it has been advised to consult other guidance documents which describe chemical, toxicological, pharmacological and pharmacokinetic properties of the medicinal substances, and provide usage instructions for special products such as herbal medicinal products containing concerning ingredients such as asarone, estragole, methyleugenol, Aristolochia species, etc. To continuously monitor the safety of herbal medicine in UK, a doctor-directed statistical survey is in place to collect post-marketing data about the herbal medicines. Actions of product recall will be triggered should there be high level of safety risk detected.

### Efficacy

As defined in the Directive 2001/83/EC, traditional use is justified by bibliographical or expert evidence showing that the medicinal product in question, or a corresponding product has been in medicinal use throughout a period of at least 30 years preceding the date of the application, including at least 15 years within the European Union countries. With the justification of traditional use, the registration procedure can be simpler and less costly without requiring the clinical efficacy data. The documentation of traditional use, if deemed sufficient, will be used as the primary evidence for its safety in specific conditions and plausible efficacy and pharmacological effects. The European herbal handbooks, sales figures and documented use in the Netherlands, as well as France, Belgium and United Kingdom can be used as reference for indicated uses in EU. Moreover, due to inherent complexity of herbal active substance and lack of clinical data, indications for herbal medicines are restricted based on evidence of long standing use evidence in THR (Permitted indications under the Directive on Traditional Herbal Medicinal Products). More specifically, permitted claims of efficacy are only suitable for minor self-limit medical claim such as symptomatic relief cold, minor self-limiting bacterial infections, minor upper respiratory infections and other minor disease. The medical claims about treating more serious diseases such as cardiovascular disease that require proven medical treatments are restricted and require strong clinical data to support their safety and efficacy.

## The US

In the US, there is no category established specifically for T&CM products. For products containing the same herbal ingredients, they may be regulated as drug, cosmetic or device depending on the route of administration, dosage form, formulation, evidence about the safety and the intended use. As a general rule, any product that contains plant materials, algae, macroscopic fungi, or combinations thereof, and is intended to be used as drug (to treat, prevent or mitigate a medical condition) is referred to botanical drug [24]. As far as structure functions claims are concerned, although these are allowed for drugs and dietary supplements, only drugs are allowed to have the structure function claims made specific to certain diseases or other medical conditions. According to the Dietary Supplement Health and Education Act (DSHEA), dietary supplements are not allowed to make any disease-related claims, neither explicitly not implicitly. Therefore, for the purpose of this study, DSHEA and other regulations bound to dietary supplements are not included for analysis.

T&CM products including TCMs containing herbal ingredients and carrying therapeutic claims are mostly considered as botanical drug product and may be marketed under either an over-the-counter drug monograph or an approved new drug application (NDA). For a botanical drug substance to be included in an OTC monograph, the safety and effectiveness must be well-established and supported with sufficient published data generated from adequate and well-controlled clinical studies. For T&CM products which do not have marketing history in the US or a foreign country, do not meet the criteria for inclusion in the OTC drug monograph based on available evidence of safety and effectiveness, nor carry a health claim appropriate for non-prescription use, the botanical drug product must undergo the NDA to obtain marketing approval from the FDA. NDA must be supported with substantial evidence of quality, safety and efficacy derived from adequate and well-controlled clinical studies. The recommendations on quality, nonclinical, clinical, and other unique aspects of botanical drug development are provided in the Guidance for Industry: Botanical Drug Development [38] and are summarized in the following.

### Quality

Botanical raw material must confirm to Good Agricultural and Collection Practices (GACPs). In terms of compliance with pharmaceutical Good Manufacturing Practices, the same requirements hold for botanical drugs and other drug products in the US as required by the assessment process of NDA. Details information about the botanical raw material control (e.g., agricultural practice and collection), identification of the medicinal plants ingredients (e.g. morphology, macroscopic and microscopic analysis, chemical analysis, or DNA fingerprinting) may be required depending on the intended use. Demonstration of consistency in terms of identity, purity and impurities such as heavy metals, aflatoxins and pesticides) of the raw materials, ingredients, and products will also be assessed.

### Safety

For non-prescription botanical drug, “absolute” safety is warranted meaning the benefits based on intended use outweighs any risks to the users despite in the absence of any professional guidance. For botanical drugs to be considered as “new” drugs, they are not generally recognized as safe and effective under the conditions prescribed, recommended, or suggested on the labeling. They are required to undergo the regulatory pathway of an Investigational New Drug (IND). Despite any long history of use, additional safety data from non-clinical and clinical stages, may be required to justify the proposed indication, route of administration or target populations. For the non-clinical safety assessment, the results of important in vitro assays in human and/or nonhuman animal tissue should be included when pharmacology/toxicology is evaluated, which includes carcinogenicity and reproductive toxicology studies. Clinical pharmacology trials are also needed to be included for ensuring the consistency of safety use, providing the findings from the pharmacokinetics and pharmacodynamics data, and exposure–response relationships which support dose selection and or dose modification. Overall exposure at appropriate doses/durations and demographics of target populations, explorations for dose response, special animal and/or in vitro testing, routine clinical testing, metabolic, clearance, and interaction workup and evaluation for potential adverse events for similar drugs in drug class must also be accessed.

### Efficacy

The evidence requirements demonstrating efficacy for botanical “new” drugs are the same as for other drugs. Substantial evidence consisting of adequate and well-controlled investigations including clinical investigations by qualified experts with scientific training and experiences is required. Consistency in safety and efficacy from batch to batch will also be assessed to address the inherent challenges of most botanical drugs. The FDA adopted a “totality-of-evidence” approach taking into consideration of the unique limitations of botanical drug products in terms of characterization of the ingredients and the end-products. Well-controlled raw materials, robust manufacturing process and quality controls including comprehensive fingerprints, clinically relevant bioassay, an multiple-dose and multiple batch clinical data are required to demonstrate the quality consistency, and thus therapeutic consistency of a botanical drug product from batch-to-batch. Up to date, the FDA has approved two NDAs for prescription botanical new drugs, both of which were complex botanical mixtures designed to become new therapies and yet met the legal requirements for drugs in the U.S.

## Discussion

Policy standards regarding market entry of T&CM products vary greatly from one country/region to another [39, 40]. This is a significant factor determining the extent that these products can be internationalized. In this study, CPMs from China were used as the product of interest to systematically compare the market entry requirements of T&CM products in Macau-China, Hong Kong-China, Singapore, Australia, Canada, the European countries and the US. It has been shown that, across the countries and regions studied, the governing authorities’ rulings on market entry of T&CM products including CPMs is primarily focused on the quality, safety and efficacy of the products. As presented in Table 1, substantial variations were identified among countries in the classification of the products, registration pathways and the document requirements for the registration dossier. This can be explained by the different interpretations of the risks and benefits of T&CM products, compounded by the unique features including complex composition and multiple mechanisms of actions [41–43].

**Table 1:** Regulation about Chinese patent medicines from China in selected countries and regions

	Countries/regions							
	Macau SAR	Hong Kong SAR	Singapore	Australia	Canada	European countries		US
						The Netherland	UK	
Possible terminologies for CPMs from China in the country/region	Chinese patent medicines	Proprietary Chinese medicines	Chinese proprietary medicines	Complementary medicines (listed) Complementary medicines (registered)	Natural Health Products	Traditional herbal medicinal products	Herbal medicine	Botanical drug product
Definition	“A product which has a definite pharmaceutical form and composes of (1) one or more ingredients from Part I and/or Part II of “List of Traditional Chinese Medicine Ingredients for consumption in Macao SAR”; or (2) one or more ingredients from Part III of “List of Traditional Chinese Medicine Ingredients for consumption in Macao SAR” and carry claims about treating, alleviating or preventing a disease or symptoms on the product label”	“Refer to any proprietary products: (a) composed solely of the following as active ingredients: (i) any Chinese herbal medicines, (ii) any materials of herbal, animal or mineral origin customarily used by the Chinese; or (iii) any medicines and materials referred to in subparagraphs (i) and (ii) respectively; (b) formulated in a finished dosage form; and (c) known or claimed to be used for the diagnosis, treatment, prevention or alleviation of any disease or any symptom of a disease in human beings, or for the regulation of the functional states of the human body	“Any medicinal product (a) which has been manufactured into a specific dosage forms and contains one or more active substances derived wholly from any plant, animal and/or mineral using active substances that are listed in the current edition of “A Dictionary of Chinese Pharmacy” or “The Chinese Herbal Medicine Materia Medica”	“Therapeutic goods consisting wholly or principally of one or more designated active ingredients, each of which has a clearly established identity and (a) a traditional use or (b) any other use prescribed in the regulations”	“Naturally occurring substances that are used to restore or maintain good health, often made from plants, but can also be made from animals, microorganisms and marine sources”	“Also referred to as phyto-therapeutic products, medicinal products whose active ingredients contain exclusively plants, parts of plants or plant materials or combinations thereof, in crude or processed form; used for at least 30 years, including at least 15 years within the EU”	“A product is a herbal medicine if the active ingredients are herbal substances and/or herbal preparations only”	“A botanical drug product is intended for use in the diagnosis, cure, mitigation, treatment or prevention of disease in humans, which consists of vegetable materials (plant materials, algae, macroscopic fungi, or combinations thereof)”
Regulatory authority	Department of Pharmaceutical Affairs, Health Bureau	Chinese Medicine Council of Hong Kong (CMCHK) Chinese Medicine Division of the Department of Health	Health Science Authority (HSA)	Therapeutic Goods Administration (TGA)	Health Canada (HC)	Medicine Evaluation Board (MEB)	Medicines and Healthcare products Regulatory Agency (MHRA)	US Food & Drug Administration (FDA)
Major legal documents	Decreta-Lei n 53/94/M Decision No. 7/SS/2004 Technical Instruction No. 02/2005	Chinese Medicine Ordinance July 1999 Section 119—registration 3rd Dec 2010	Medicine Act	Therapeutic Goods Act 1989	Natural Health Products Regulations of the Food and Drugs Act	Traditional Herbal Medicinal Products Directive (2004/24/EC); the Dutch Medicines Act; and relative guidelines	Directive 2004/24/EC for THR (Traditional Herbal Registration) Directive 2001/83/EC for MA conventional)	Federal Food, Drug, and Cosmetic Act
Requirements to demonstrate quality	Manufacturer’s license, GMP standards recommended but not mandated	GMP standards and QC tests	GMP standards PIC/S guide	GMP standards PIC/S guide and QC tests	GMP standards and QC tests	GMP standards and QC tests	GMP standards and QC tests	GMP standards and QC tests
Requirements to demonstrate safety	Drug Registration Certificate issued by the competent authority in the country of manufacture or country of origin Limits of heavy metal content (Technical Instruction No. 2/2003) Microbiological limits (Technical Instruction No. 1/2004) Additional requirements for CPMs containing bovine-derived ingredients (Chief Executive Decision No. 120/2005 and Technical Instruction No. 02/2001)	Heavy metals and toxic elements test report, pesticide residues test report, microbial limit test report, acute toxicity test report, long-term toxicity test report, and the summary report on product safety documents	Declaration on the absence of any poisons as defined in the Poisons Act (Cap. 234); Endorsement by manufacturer that product does not contain any Western drugs or active synthetic substances; TSE undertaking for products containing materials derived from ruminants; Information about for fermented substance for products containing fermented substance(s)	Contains ingredients on the TGA’s list of permissible ingredients only for listed complementary medicines Toxicological tests for registered complementary medicines	Conforming any of the monographs, “benefit to risk” assessment, additional evidence whenever needed	Bibliographic review of safety data together with an expert report”; no need for safety test; clinical safety data (supportive)	Bibliographic data for THR Toxicological tests for MA	Non-clinical safety assessment: pharmacology/toxicology tests; clinical pharmacology trials
Requirements to demonstrate efficacy		The materials of Interpretation and principle of formulating a prescription (excluding granules), reference materials on products efficacy and the summary report on product efficacy documents Extra documents (principal pharmacodynamic studies report, general pharmacological studies, clinical trial protocol and summary report) maybe required	TCM system of therapeutics, historical records and traditional uses in pharmacopeias or with reference to A Dictionary of Chinese Pharmacy and The Chinese Herbal Medicine Materia Medic	Traditional evidence and available efficacy data for listed complementary medicines Clinical studies for registered complementary medicines	Attestation to a monograph for Class I category (low risk); monograph and additional information such as phase 2 clinical trials for Class II category (“medium risk”); high level of evidence from controlled clinical trials for Class III category (“high risk”)	Justification of traditional use [long tradition of use for at least 30 years (including 15 years in the EU)]; bibliographical or expert evidence Clinical studies for MA	Restricted indications and long tradition of use for at least 30 years (including 15 years in the EU) for THR Clinical studies for MA	Individual studies/clinical trials

With regards to quality, the minimal international consensus is that all T&CM products must meet certain quality standards that demonstrate authentication, identification and chemical composition of the products. All the countries/regions reviewed in this study require manufacturing sites of T&CM products to follow GMP and/or PTC/s Guide, thereby, ensuring that the products are of consistent quality and are safe for use by consumers. Special emphasis on quality and therapeutic consistency from batch to batch was noted in some regulatory systems such as the FDA. The only exception to these quality requirements is Macau where there is no registration system for TCMs in place. In terms of safety, some countries/regions hold a list of ingredients considered safe for use, or require the attestation to the published monographs as a minimum, whilst others simply have a banned-for-use system. Most of the countries/regions in this study clearly specify the need for certificates of analysis that include tests for microbial, pesticide residues, and heavy metals and other harmful substances in all T&CM products. Respect and recognition of historical traditional use and knowledge is an important consideration adopted by most of the countries studied in their regulatory framework for supporting the efficacy and safety of T&CM products.

While marketing as “medicine” is important to preserve the therapeutic values of T&CM products, there is a growing trend to market eligible T&CM products as “non-medicine” products such as health care products, functional food or food supplements, depending on the terminology adopted in different countries. For TCMs supported with a long history and high prevalence of use, the international market potential and expansion into the non-drug category represents a promising route for TCMs to achieve sustainable development through internationalization. Criteria of market entry of T&CM products (with TCMs in particular) as non-drug entities will be worth exploring to further inform potential strategies for sustainable development of T&CM products and the internationalization of TCMs in future studies.

There were some limitations to this study. The sample size of the countries/regions included in this study is insufficient to fully demonstrate the possible variations of the current regulatory approach towards T&CM products around the world. However, the regulatory systems analyzed in this study does represent a wide spectrum of regulatory enforcement. The degree of variations observed in this study does raise awareness about one of the biggest challenges facing the internationalization of TCMs.

## Conclusion

The market entry requirements for T&CM products for Macau-China, Hong Kong-China, Singapore, Australia, Canada, the European countries and the US were analyzed and compared with the major differences highlighted. The different evaluation standards adopted by these regulatory authorities pose a number of barriers and opportunities for the internationalization of T&CM products and have great implications for internationalization of TCMs. In order to assure the quality, safety and efficacy of T&CM products, it is important for the sponsors and the regulators to follow closely the developments evolving around the regulation of these products.

## Abbreviations

TCMs: traditional Chinese medicines
CPMs: Chinese patent medicines
RCTs: randomized controlled trials
T&CM: Traditional and Complementary Medicine
WHO: World Health Organization
SAR: special administrative region
pCms: Proprietary Chinese medicines
ARTG: Australian Register for Therapeutic Goods
HSA: Health Science Authority
Cpm: Chinese proprietary medicines
THR: traditional herbal registration
MA: marketing authorization
MHRA: Medicines & Healthcare products Regulatory Agency
MEB: Medicines Evaluation Board
GMP: Good Manufacturing Practices
